# Move more for life: the protocol for a randomised efficacy trial of a tailored-print physical activity intervention for post-treatment breast cancer survivors

**DOI:** 10.1186/1471-2407-12-172

**Published:** 2012-05-08

**Authors:** Camille E Short, Erica L James, Afaf Girgis, Patrick Mcelduff, Ronald C Plotnikoff

**Affiliations:** 1 School of Medicine and Public Health, Priority Research Centre for Health Behaviour, Priority Research Centre for Physical Activity and Nutrition, University of Newcastle, Callaghan, Australia; 2 Ingham Institute for Applied Medical Research, South Western Sydney Clinical School, University of New South Wales, Sydney, Australia; 3 Clinical Trials Unit, Hunter Medical Research Institute, School of Medicine and Public Health, University of Newcastle, Callaghan, Australia; 4 School of Education, Priority Research Centre for Physical Activity and Nutrition, University of Newcastle, Callaghan, Australia

## Abstract

**Background:**

Due to early detection and advances in treatment, the number of women surviving breast cancer is increasing. Whilst there are many positive aspects of improved survival, breast cancer survival is associated with many long-term health and psychosocial sequelae. Engaging in regular physical activity post-diagnosis can reduce this burden. Despite this evidence, the majority of breast cancer survivors do not engage in regular physical activity. The challenge is to provide breast cancer survivors with appealing and effective physical activity support in a sustainable and cost-effective way. This article describes the protocol for the *Move More for Life* Study, which aims to assess the relative efficacy of two promising theory-based, print interventions designed to promote regular physical activity amongst breast cancer survivors.

**Method and design:**

Breast cancer survivors were recruited from across Australia. Participants will be randomised into one of three groups: (1) A tailored-print intervention group, (2) a targeted-print intervention group, or (3) a standard recommendation control group. Participants in the tailored-print intervention group will receive 3 tailored newsletters in the mail over a three month period. Participants in the targeted-print group will receive a previously developed physical activity guidebook designed specifically for breast cancer survivors immediately after baseline. Participants in the standard recommendation control will receive a brochure detailing the physical activity guidelines for Australian adults. All participants will be assessed at baseline, and at 4 and 10 months post-baseline. Intervention efficacy for changing the primary outcomes (mins/wk aerobic physical activity; sessions/exercises per week resistance physical activity) and secondary outcomes (steps per day, health-related quality life, compliance with physical activity guidelines, fatigue) will be assessed. Mediation and moderation analyses will also be conducted.

**Discussion:**

Given the growing number of cancer survivors, distance-based behaviour change programs addressing physical activity have the potential to make a significant public health impact.

**Trial registration:**

Australian New Zealand Clinical Trials Registry (ANZCTR) identifier: ACTRN12611001061921

## Background

Due to earlier detection and advances in treatment, more and more women are surviving breast cancer each year [1]. Whilst improved survival is duly welcomed, breast cancer survivors are faced with both short and long-term health and psychosocial sequelae [2], including fatigue, reductions in physical and cognitive functioning, reductions in bone health, lymphedema, weight gain and mood disturbances [3-6]. Compared to the general (non-cancer) population, breast cancer survivors are at an increased risk of co-morbid chronic conditions and death from both cancer and non-cancer causes [7]. As such, there is a growing need for effective cancer recovery services that can help to improve the quality of life of breast cancer survivors and negate the associated health burdens and risks [8].

One promising cancer recovery strategy is the promotion of regular physical activity (PA) [9,10]. Evidence from health outcome trials suggests that regular PA can address both the psychological and physiological burdens presented after breast cancer diagnosis and treatment [11,12]. Furthermore, observational research suggests that regular PA may also have an impact on survival, with breast cancer survivors who are active after treatment having a lower risk of cancer recurrence, co-morbidities and death from all causes compared to those who are less active, regardless of cancer stage [13-15]. In recognition of these benefits, detailed exercise prescription guidelines for cancer survivors have been published by professional bodies in both Australia and North America [9,10,16].

There is also new evidence that addressing the pattern of activity is important, with unique metabolic consequences associated with prolonged sedentary behaviour, regardless of total activity time [15,17]. Despite this evidence, the majority of breast cancer survivors are not sufficiently active for health [17,18] and efforts to encourage regular PA and reductions in sitting time are not a routine part of the cancer treatment or rehabilitation process [19-22].

Whilst over 70 PA intervention studies have been conducted with cancer survivors, the majority have been atheoretical face-to-face programs conducted during the treatment phase [23-26]. Whilst these interventions have been efficacious in improving important outcomes for cancer survivors, there is a need for more sustainable, less resource intensive approaches that can support survivors beyond the initial treatment phase [27-29]. Such programs should be grounded in behaviour change theory, and address the unique determinants of PA adoption and maintenance in the post-treatment breast cancer population [30,31].

The purpose of this study is to evaluate the relative efficacy of two promising distance-based approaches (targeted and tailored print interventions) for promoting PA among post-treatment breast cancer survivors compared to a standard recommendation control group. In targeted-print interventions, irrelevant information is reduced by providing individuals with materials targeted to a particular subgroup they belong to (e.g., breast cancer survivors) [32]. In tailored-print interventions, computer technology is utilised to provide individuals with personalised advice based on information specific to them (derived from individual assessment) [32]. Both approaches have been put forth as low-cost, evidence-based alternatives to resource intensive face-to-face programs [32,33], but little information exists about the relative efficacy and the cost/benefit of these approaches in the physical activity domain.

Some theories of information processing, such as The Elaboration Likelihood Model [34], suggest that people are more likely to process information in a way that is conducive to behaviour change, if it is personally relevant to them. Based on this model, we hypothesise that individuals randomised into either the targeted or tailored print groups will experience significantly greater improvements at each timepoint on all primary and secondary outcomes compared to the standard recommendation control group. Furthermore, given the greater level of personalisation of materials in the tailored-print condition, we expect participants in the tailored-print group to experience greater improvements across PA outcomes compared to participants in the targeted-print group. This hypothesis relies on the assumption that breast cancer survivors are a somewhat heterogeneous group in terms of determinants (i.e., demographics, social-cognitive and ecological factors) of PA behaviour change.

## Methods

### Design

**Figure 1:**
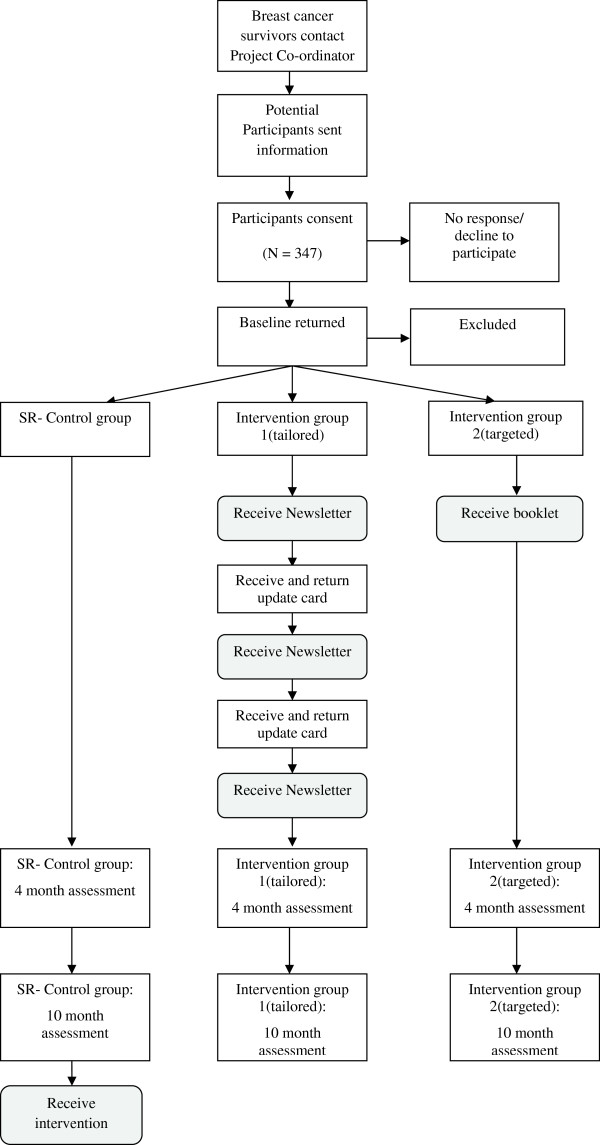
Study flow chart.

This study is a nationally-based, three-arm randomised controlled trial (RCT), testing the relative efficacy of two distance-based PA interventions (tailored and targeted print) compared to a standard recommendation control group. Participants will complete data collection at baseline, 4 months and 10 months. Ethics approval was obtained from the University of Newcastle Human Research Ethics Committee (H-2010-11-3). The RE-AIM framework [35] will serve to guide the dissemination of this program in terms of adoption, implementation and maintenance. The conduct and reporting of this study will adhere to the Consolidating Standards of Reporting Clinical Trials (CONSORT) guidelines [36] and to the Reporting Standards for Studies of Tailored Interventions [37]. The study flow chart is presented in Figure 1 (note: recruitment for this study is complete). 

### Participants

#### Selection criteria

Female breast cancer survivors who are over the age of 18 and who have finished “active” cancer treatment (defined as surgery, chemotherapy and/or radiotherapy), who can read and write in English were eligible to participate in the study. All potential participants were required to complete a physical activity readiness questionnaire (PAR-Q; [38]), to screen participants and identify those requiring review from their doctor prior to study enrolment.

#### Recruitment

Participants were recruited using convenience sampling methods from a range of sources across Australia. Specifically, this involved (1) asking organisations (e.g., The Breast Cancer Network Australia, The Cancer Council, YWCA Encore) and health professionals (e.g., breast care nurses) with direct contact with breast cancer survivors to disseminate information about the study on behalf of the research team; (2) promoting the study at events potentially relevant to eligible participants (e.g. breast cancer forums); and (3) snowballing recruitment (inviting participants to pass on study information to potentially eligible friends and acquaintances).

### Randomisation

The randomisation sequence will be generated by a statistician (PM) using SAS 9.2 statistical software. An equal number of participants will be randomised to each group (1:1:1) using a randomised block design, with a block size of six, to ensure the study groups are balanced [39,40].

Participants, identified only by their ID number, will be randomised by an administrative staff member into groups upon receipt of their baseline survey. All project team members will be blinded to this process and participant details will remain de-identified until participant allocation is completed. Participant blinding is not possible due to the difference in delivery schedule of the two interventions.

### Statistical power and sample size

The study’s primary analysis will be the comparison of self-reported PA behaviour (i.e. mins/week of aerobic exercise and sessions/week of resistance training) between the three groups, from baseline to the 4 month time-point. Assuming a small-moderate correlation (r = 0.4) between baseline and post-intervention, to detect a mean difference of 0.5 standard deviation between study groups (small-medium effect size) for the main dependent outcome (i.e., PA behaviour) at post-intervention [41] the required sample size is 100 participants per group, allowing for a 20% loss to follow-up (power = 0.80; alpha = 0.01). An alpha of 0.01 was used to adjust for multiple comparisons.

As a secondary consideration, we also ensured that this sample size would be adequate to detect a clinically significant change in step counts per day (2000 steps per day [42], standard deviation of 3500 [43]) and found that we would be adequately powered to detect meaningful changes in both self-report and objective PA outcomes.

### Outcomes

A pen-and-paper questionnaire is completed at baseline, 4 months post baseline (immediate post- tailored intervention follow-up), and 10 months post-baseline (7 month post tailored-intervention follow-up). At each of these time-points, participants will be asked to wear a pedometer for seven days and complete a step count diary.

#### Primary outcome

The primary outcome variables, minutes of PA (aerobic) per week and average number of sessions/exercises per week (resistance) will be assessed using an adapted version [44] of the validated Godin Leisure-Time Exercise Questionnaire (GLTEQ) [45]. The adapted version will incorporate a resistance training (RT) measure [44,46] that asks participants to report the frequency (times per week) and duration (average times per session) of resistance training activities on average over the past month. The original measure has been found to be both reliable and valid [47].

#### Secondary outcomes

##### Step counts

Average daily step counts [48] will be estimated based on at least three days of pedometry, which is sufficient to reliably estimate pedometer-determined PA [49,50]. Participants will be instructed to zero the pedometer and record their accumulated steps at the end of each day for seven days, using the step count diary provided. The step count diary will also be used to record instances where the pedometer was intentionally removed (e.g. swimming) or when the participant forget to wear the device. Where reported, step count equivalents for non-ambulatory activities (e.g. swimming, cycling) will be calculated and added to the step count total using the method outlined by Miller et al. [51].

##### Adherence to PA guidelines

PA type, frequency and duration measured by 8 items from the adapted version of the GLTEQ [44,46] will be used to calculate whether or not participants are meeting the PA guidelines for cancer survivors [10].

##### Sedentary behaviour

Sedentary behaviour is measured using a validated five item scale asking about time spent sitting (hours and minutes) each day during the week and on the weekend in the following situations (a) while travelling to and from places; (b) while at work; (c) while watching television; (d) while using a computer from home; and (e) in leisure time not including watching television (e.g. visiting friends, dining out) [52].

##### Health related quality of life

Quality of life is measured using version 4 of the internationally validated 37-item FACT-Breast measurement system (FACT-B) [53]. The FACT-B is multidimensional, consisting of subscales measuring cancer specific aspects of physical well-being, emotional well-being, social well-being, functional well-being, and 10-items measuring breast cancer specific concerns.

##### Fatigue

Fatigue is measured using the validated 13-item FACIT (Functional Assessment of Chronic Illness Therapy) Fatigue scale, which assesses self-reported tiredness, weakness and difficulty conducting usual activities [54].

#### Social cognitive mediators of physical activity

Hypothesized social cognitive mediators of PA behaviour are assessed using previously published, validated instruments where possible. Some items were adapted to make them more appropriate for use in this study. The adaptations were based on our own qualitative research and formative research in the field (e.g. [55]) and were tested for face validity using a small convenience sample (n = 5) of post-treatment breast cancer survivors. In each survey, the time referent used for the items is framed based on the timing of the proceeding follow-up survey (i.e. the baseline survey time referent is “the past/next four months” and the four and ten month follow-up surveys time referent is “the past/next six months”).

##### Outcome expectations

Outcome expectations is measured using 5 general items from the validated exercise pros subscale [56] with 6-additional items developed for this study based on formative research among breast cancer survivors [55,57], including our own qualitative research and information provided by experts in the field. The items in the scale assess the extent that individuals agree or disagree (1 = strongly disagree to 5 = strongly agree) that participating in regular PA over the next 4 month would for them: reduce tension or manage stress; increase confidence about one’s health; help to sleep better; have a more positive outlook; help control weight; regain lost strength; prevent a cancer recurrence; be enjoyable; increase fatigue; increase joint pain or result in lymphoedema. An example item includes “Over the next four months, participating in regular PA will help me prevent a cancer recurrence.”

##### Outcome expectancies

Outcome expectancies will be assessed by asking participants to rate how important each of the outcome expectations are to them (e.g. “For me, reducing joint pain is”) on a 3-point scale (1 = unimportant; 2 important; 3 very important). This scale has been utilised and tested in prior research [44,58].

##### Self efficacy

Task self-efficacy will be assessed using 4-items developed [57] and evaluated [59] in previous studies with breast cancer survivors and 3 additional items developed for this study to assess task-self efficacy for resistance training activities. The items assess the participant’s level of confidence (1 = not at all confident to 5 = extremely confident) that over the next 4 months they can: walk for 20 minutes without stopping; jog for 10 minutes without stopping; climb 3 flights of stairs; exercise for 20 minutes at a level hard enough to cause an increase in heart rate; do 6 wall push ups in a row; do one small session of resistance training including 6 different exercises; and do yoga for 60 minutes (Example item: “Over the next four months, I can do 6 wall push ups in a row”).

Barrier self-efficacy will be assessed using 12-items based on previous scales used in chronic disease populations (7 items developed and tested by Rogers’ et al. [59] among breast cancer survivors and 5 items developed and tested by Plotnikoff et al. among diabetes patients [56,60]) and one item (“when I can’t notice any improvements in my body”) developed for this study based on formative research. Participants will be asked to rate their confidence (1 = not at all confident to 5 = extremely confident) that they can participate in regular PA over the next four months when: they lack the discipline to exercise; exercise is not a priority; the weather is bad; feeling tired; lack time; do not enjoy exercising; do not have someone to encourage them to exercise; in a bad mood or feeling depressed; have to do it alone; can’t notice any improvements in fitness; can’t notice any improvements in body; feel stiff and sore; and feel ill.

##### Behavioural capability

Behavioural capability is measured using 6-items assessing specific components of PA knowledge and skill that were developed for this study. Participants will be asked to rate on a 5-point likert scale (1 = strongly disagree to 5 = strongly agree) how much they agree with each of the statements: I know how to warm up and cool down before/after an exercise session; I have a good idea of what type of PA to do to gain health benefits; I have a good idea of how hard I should engage in PA to gain health benefits; I have a good idea of how much PA I should do to gain health benefits; I have the skills I need to engage in aerobic physical activities; and I have the skills I need to engage in resistance-based physical activities.

##### Environment

*Social support* is assessed using the 15-item social support for exercise habits scale [61]*.* Participants are asked to rate how often during the past four months their friends and family (separately) supported them/discouraged them to exercise in a variety of ways. Response options range from 1 (none) to 5 (very often). An example item is: “During the past four months, my friends gave me encouragement to stick with my exercise program”. *The perceived built environment* will be assessed using an adapted version of the 7-item IPAQ environmental module [62]. Participants will be asked to rate how much they agree or disagree (1 = strongly disagree to 5 = strongly agree) with the following statements: most of the houses in my neighbourhood are detached houses; many shops, stores, markets or other places to buy things I need are within easy walking distance of my home; my home is within a 10-15 minute walk to a bus or train station; there are footpaths on most of the streets in my neighbourhood; there are facilities to bicycle in or near my neighbourhood; my neighbourhood has several free or low cost recreation facilities; and the crime rate in my neighbourhood makes it unsafe to go on walks at night.

##### Self control and performance

*Self-regulation* will be assessed using a 12-item scale developed for use among older adults [63]. The items measure six subscales of self-regulation (self-monitoring, goal setting, eliciting social support, reinforcements, time management, relapse prevention) and can be combined to produce an overall score [63]. An example item is “Over the past 4 months, how often did you rearrange your schedule to ensure you had time for physical activity”. Response options range from 1 (never) to 5 (very often).

*Action planning* will be assessed using 4-items developed by Rise et al. [64] and adapted by Rhodes et al. [65](to say “physical activity” instead of “exercise”). Participants will be asked to rate the following statements according to their plans over the next two weeks (1 = no plans to 5 detailed plans): I have made plans concerning ‘when’ I am going to engage in regular PA; I have made plans concerning ‘where’ I am going to engage in regular PA; I have made plans concerning ‘what’ kind of regular PA I will engage in; I have made plans concerning ‘how’ I am going to get to a place to engage in regular PA.

#### Socio-demographics

The following socio-demographic data will be collected: date of birth, marital status, parental status, living arrangement, country of birth, education, employment, income, internet access, health insurance status and geographical location.

#### Health status and cancer history

At baseline and each follow-up time point (where applicable), participants are asked five questions about their health status (physical limitations, perceived weight, menopause status, co-morbidities) and nine questions about their cancer diagnosis (age at diagnosis, cancer stage, treatment type, and prognosis).

#### Process evaluation

Participant evaluation of the intervention materials will be measured using 15 multiple choice items and one open-ended question, included in the immediate post intervention follow-up questionnaire. The 15 multiple choice items were purpose-designed by the research team and are based on the Elaboration Likelihood Model (ELM) [34], which is often utilised to explain the effects of health communication interventions. An example item includes: “how personally relevant was the health information you received” (1 = not at all relevant to 5 = very relevant). The open ended question provides participants with a chance to make comments about the intervention materials.

#### Procedure

Potential participants were asked to contact the project co-ordinator to express their interest in participating in the study. Potential participants were then provided with an information statement and a consent form and asked to return it to the project team within two weeks. Information was resent at two weeks if no response was received.

Participants will be asked to complete a pen-and-paper questionnaire, wear a pedometer for seven days and complete a pen-and-paper step count diary at baseline, four and ten months from baseline (Figure 1). Participants will be instructed to return the pedometer with the written materials using a reply paid envelope as soon as possible after each assessment period is complete. Participants who do not return the baseline questionnaire, and step count diary and pedometer within two weeks will receive one reminder call from the project co-ordinator. Participants who do not return the assessment materials within three weeks after this reminder call will be excluded from the trial.

Upon receipt of the baseline questionnaire, an administrative assistant will allocate participants using the ID number written on the questionnaire into one of three groups using the randomly generated allocation sequence provided by the statistician. Participants will be sent intervention or standard recommendation materials within three weeks of allocation.

Participants in the tailored-print intervention group will be sent additional intervention materials at 6 weeks and 12 weeks post baseline and update cards (3-item update card), 4 weeks and 8 weeks post base-line and asked to return them to the research team using a reply paid envelope within 7 days. Participants in the standard recommendation group will receive one tailored newsletter and a pdf version of the targeted guidebook after completion of the10 month follow-up survey (Figure 1).

#### Interventions

##### Targeted-print intervention

Participants randomised into this group will receive a copy of a theory-based exercise guidebook developed specifically for promoting physical activity among breast cancer survivors. This guidebook was developed for use and evaluated in a previous study [66] and has been described in detail elsewhere [67]*.* We made minor changes to the guidebook to adapt it to an Australian audience (e.g. substituting photos and text relating to snow).

##### Tailored-print intervention

Participants randomised into this group will be mailed three Social Cognitive Theory-based [68,69] computer-tailored newsletters over a 12 week period (6 weeks apart). Each newsletter will be four A4 pages in length and will provide advice and feedback unique to the individual that relates to key determinants of PA adoption and maintenance among breast cancer survivors (as stipulated by previous research in the field [57,70-77] and Social Cognitive Theory [68,78]). The advice participants receive will be tailored using information derived from individual assessments at baseline, and ‘update cards’ (assessing PA and goal setting behaviour over the last month) sent to participants (in this group only) at 4 weeks and 8 weeks post-baseline. In each case, participants will be mailed the tailored-newsletters within two weeks after the completed assessment is received (see Figure 1).

*Newsletter 1 (N1)* will include information on the Australian PA guidelines for cancer survivors (non-tailored), tailored feedback on PA behaviour (aerobic, resistance and sitting time) relative to the guidelines, information about the beneficial outcomes of PA, safety advice and an action planning activity. An activity planner and exercise illustrations (stretches and resistance training exercises) will also be included.

*Newsletter 2 (N2)* will include expert advice from a behaviour change expert (non-tailored), feedback on PA performance (aerobic, resistance and sitting time) relative to N1, a testimonial illustrating success, advice on eliciting social support and an action planning activity.

*Newsletter 3 (N3)* will include expert advice from an exercise physiologist (non-tailored), tailored feedback on PA performance (aerobic, resistance and sitting time) relative to N2 and N1, advice on restructuring the physical environment, information about available support services and an action planning activity. See Additional file 1 (Table S1) for a brief overview of how Social Cognitive Theory was operationalised to form these intervention strategies and what variables were used to tailor information.

##### Standard recommendation control group

Participants randomised into this condition will receive the “An active way to better health” brochure published by the Australian government, detailing the national physical activity guidelines for adults [80]. The guidelines stipulate that Australian adults should: (1) think of movement as an opportunity; (2) be active every day in as many ways as you can; (3) do 30 minutes of moderate intensity physical activity on most, preferably all days; and (4) if manageable, do vigorous activity for extra health benefit. A copy of the brochure can be downloaded free of charge from http://www.healthyactive.gov.au.

#### Statistical analysis

Analyses will be conducted according to the intention to treat principal, as outlined by White et al. [81]. Namely, the primary analysis will be conducted using all observed data (i.e., a completers analysis) and sensitivity analyses (accounting for all randomised participants) will be conducted to explore the impact of missing data [81]. Differences between treatment groups in the primary outcome measures (i.e. the two PA scores) 4 months after randomisation will be tested using Analysis of Covariance (ANCOVA). The outcome in the model will be the subjects physical activity score at 4 months and the predictors will be treatment group and baseline value of the physical activity score. In the analysis of each of the two PA measures, if the p-value for the treatment group is less than 0.025 (adjusted to account for the two primary PA analyses) then post hoc tests of the 3 pair wise comparisons will be undertaken to determine which treatment groups are different. Socio-cognitive and QOL measures will also be analysed using ANCOVA models. The study’s primary analysis will be the comparison of PA behaviour between the three groups, from baseline to the 4 month time-point. Secondary analyses will examine the PA behaviour change between the study groups across the other study time-point (i.e., 10 months). A mediation analysis on the employed social-cognitive variables will also be conducted to explore the causal mechanism of any intervention effects. Planned subgroup analyses include age, PA status at baseline, time since treatment, BMI, built environment, and co-morbidity status.

## Discussion

This study will test the relative efficacy of two theory-based PA behaviour change interventions. In doing so, this study will address a seminal research question in distance-based patient-centred care – is tailoring or targeting health education messages a more efficacious approach to health behaviour change in the PA domain? Furthermore, this study will be one of the first to promote a pattern of PA that addresses the metabolic consequences of unbroken sedentary behaviours and the advantages of completing both aerobic and resistance-training exercises. The limitations reported in previous research will be addressed by examining adherence after the intervention period and by utilising an objective measure of PA behaviour (i.e., pedometers). Finally, this study will add to the behaviour change literature by addressing the paucity of knowledge surrounding determinants of PA behaviour change among cancer survivors and potential mediators of intervention effects.

## Competing interests

The authors declare that they have no competing interests.

## Authors’ contributions

ELJ and CES conceived the study. CES, AG and RCP obtained the funding. All authors provided input into the study design. CES was primarily responsible for intervention design and recruitment, with significant input from ELJ and RCP. CES, ELJ and RCP were responsible for drafting the manuscript. PM provided statistical guidance and support and drafted the statistical analyses section of the manuscript. All authors critically evaluated the article for content and approved the final version.

## Pre-publication history

The pre-publication history for this paper can be accessed here:

http://www.biomedcentral.com/1471-2407/12/172/prepub

## Supplementary Material

Additional file 1 **Table S1.** Operationalisation of SCT constructs for the Move More for Life intervention. Click here for file
