# Supplementary material for: Move more for life: the protocol for a randomised efficacy trial of a tailored-print physical activity intervention for post-treatment breast cancer survivors
Source: BMC Cancer. 2012 May 8;12:172. doi: 10.1186/1471-2407-12-172 (PMC3518246; doi:10.1186/1471-2407-12-172)
Supplement: Additional file 1 — Table S1. Operationalisation of SCT constructs for the Move More for Life intervention. [file 1471-2407-12-172-S1.doc]

Table 1. Operationalisation of SCT constructs for the Move More for Life intervention.

| **Newsletter 1** | | |
| --- | --- | --- |
| **Strategy** | **SCT construct** | **Tailoring variables** |
| Advice for meeting the PA guidelines for cancer survivors | Self-efficacy | PA status  PA level prior to diagnosis |
| Information about the beneficial outcomes of PA | Outcome expectations | Outcome expectancies (outcomes valued by individual) |
| Advice on exercising safely | Self-efficacy  Behavioural capability | Behavioural capability  Health status (inc Lymphedema, fatigue, co-morbidities, bone pain). |
| Action planning | Self-efficacy  Self-control and performance | PA status  PA preference |
| **Newsletter 2** | | |
| **Strategy** | **SCT construct** | **Tailoring variables** |
| Expert advice – behaviour change expert | Self-control and performance  Self-efficacy  Reinforcements | n/a |
| Feedback on PA performance | Self-efficacy  Self-control and performance | PA status  PA progress since N1 |
| Testimonial | Observational learning  Outcome expectations  Self efficacy | Self efficacy  Height  Weight |
| Advice on enhancing social support | Environment | Social support  PA preference |
| Action planning | Self-efficacy  Self-control and performance | Goal setting behaviour after N1  PA status |
| **Newsletter 3** | | |
| **Strategy** | **SCT construct** | **Tailoring variables** |
| Expert advice – exercise physiologist | Self-efficacy  Behavioural capability  Environment | n/a |
| Feedback on PA performance | Self-efficacy  Self-control and performance | PA status  PA progress since N1 and N2 |
| Tips on changing PA environment | Environment  Self-control and performance | Access to PA facilities  Work status |
| Information on gaining further support | Environment | Internet access  Geographical location (State) |
| Action planning | Self-efficacy  Self-control and performance | Goal setting behaviour after N1 and N2  PA status |
